# Enhanced superconducting transition temperature in electroplated rhenium

**DOI:** 10.1063/1.5027104

**Published:** 2018

**Authors:** D. P. Pappas, D. E. David, R. E. Lake, M. Bal, R. B. Goldfarb, D. A. Hite, E. Kim, H.-S. Ku, J. L. Long, C. R. H. McRae, L. D. Pappas, A. Roshko, J. G. Wen, B. L. T. Plourde, I. Arslan, X. Wu

**Affiliations:** 1 National Institute of Standards and Technology, Boulder, Colorado 80305, USA; 2 CIRES, University of Colorado, Boulder, Colorado 80309, USA; 3 Department of Physics, University of Colorado, Boulder, Colorado 80309, USA; 4 Department of Physics, University of Nevada Las Vegas, Las Vegas, Nevada 89154, USA; 5 Center for Nanoscale Materials, Argonne National Laboratory, Lemont, Illinois 60439, USA; 6 Department of Physics, Syracuse University, Syracuse, New York 13244, USA

## Abstract

We show that electroplated Re films in multilayers with noble metals such as Cu, Au, and Pd have an enhanced superconducting critical temperature relative to previous methods of preparing Re. The dc resistance and magnetic susceptibility indicate a critical temperature of approximately 6 K. The magnetic response as a function of field at 1.8 K demonstrates type-II superconductivity, with an upper critical field on the order of 2.5 T. Critical current densities greater than 10^7^ A/m^2^ were measured above liquid-helium temperature. Low-loss at radio frequency was obtained below the critical temperature for multilayers deposited onto resonators made with Cu traces on commercial circuit boards. These electroplated superconducting films can be integrated into a wide range of standard components for low-temperature electronics.

Development of superconducting materials with increased critical temperatures and practical integration into circuits and interconnects is important for many low temperature applications and experiments. Examples include high-speed, superconducting classical computers at liquid-helium temperatures, i.e., *T* ~ 4 K, where the main goal is to improve energy efficiency^1–6^ and also large-scale quantum information systems^7,8^ at ultra-low temperature, *T* < 30 mK. For the ultra-low temperature systems, i.e., work involving adiabatic demagnetization and dilution refrigerators, it is important to have superconducting technology that is easily integrated into the many dc bias and radio-frequency (rf) drive lines. This will minimize dissipation to reduce heat load on the cryostats and help in preservation of quantum information as it propagates through the system.

To that end, there is a small set of superconductors that are widely used at 4 K and to bridge the gap to ultra-low temperatures. These include Nb and its binary and ternary alloys such as Nb-N, Nb-Ti, and Nb-Ti-N. These materials are useful in terms of high critical temperature, can be used in bulk or deposited as thin films, and can be connected using ultrasonic-wire bonding techniques. However, they tend to be difficult to work with mechanically and have poor soldering properties due to strong oxidation. While work arounds to these problems exist, they are not easily integrated into standard circuit fabrication, which typically employs primarily Cu and Au electroplated parts.

Other superconductors, e.g., Al, Pb, In, Sn, Re, and high critical temperature (*T*_*c*_) oxides, are useful in many applications but have limitations due to low *T*_*c*_, low melting point, difficulty making connections, or other problems. One element in the latter category, Re, stands out as a promising candidate. Re is a transition metal that is resistant to oxidation, with a melting temperature of 3186°C.^9^ It is used widely in various industrial and scientific applications such as strengthening materials and in high-temperature thermocouples. Crystalline Re is a type-I superconductor with *T*_*c*_(Re) ≈ 1.7 K.^10^ Under strain, Re changes to a type-II superconductor and *T*_*c*_(Re) can be enhanced to about 3K.^10–12^ This is similar to effects seen in Re doped with W and Os.^13^ Moreover, Chu points out in Ref. 13 that there is a singularity close to the Fermi energy, *E*_*F*_, which could drive anomalous behavior in the superconducting transition temperature through alloying, and it has been noted that sputtered Re-Mo alloys have *T*_*c*_ up to 13 K.^14,15^ While both Re-Mo and epitaxially grown Re can be used for low-loss rf resonators in qubit circuits, they have no specific advantage over other traditional materials in that application.^16,17^

On the other hand, it has been known for a long time that Re can be electroplated in aqueous solution compatible with noble metal plating.^18–23^ In this process, the Re is deposited at the cathode in over-potential conditions, resulting in the concurrent production of hydrogen (H) in the plating region. This brings up the question of whether H or strain can be incorporated into the Re through electroplating and how that might affect its superconducting properties.^24^

Here, we report on the superconducting nature of electroplated Re including electrical resistance, critical current, magnetization, and rf loss. We prepare the Re in multilayers with noble metals because carbonaceous deposits tend to form on bare Re films. Cu, Au, and Pd were used in order to see if there were element-specific interface effects. Re films grown on both Au/Si and Cu-clad circuit-boards were studied. Type-II superconducting behavior is obtained with high critical temperatures and critical current densities and low rf loss.

The Re was deposited using a dc power supply in constant-current mode at 8A/dm^2^ from an aqueous solution containing 11g/L of KReO_4_ with the pH adjusted to 0.9 using sulfuric acid, according to the recipe in Ref. 18. The deposition time was used to control the layer thickness. The anodes were platinized titanium. Moderate stirring was done with a magnetic bar coated with polytetrafluoroethylene (PTFE) on a standard laboratory hot-plate stirrer at temperatures of 25–30 °C. Under these conditions, the Re deposits were shiny and smooth, typically dark gray, with good adhesion. The various sample parameters are reported in Table I.

To begin the study, we first grew a reference, Sample 1, of Au/Re/Au/prime-Si in vacuum using e-beam evaporation for the Au and sputter deposition for the Re. This sample was used to compare against standard Re preparation methods.

Electroplated bilayers, trilayers, and multilayers of Re and Au were then grown on vacuum-prepared Au/Si substrates, Samples 2–4. A 20 nm seed layer of electroplated Au was typically applied before the Re. Multilayers of Re/Cu, Re/Au, and Re/Pd were also electroplated directly onto commercial Cu/PTFE circuit boards, Samples 5–7. The boards were also patterned with resonators in order to test the rf properties of the multilayers. PTFE was used for its low loss tangent, tan *δ* = 6.8 × 10^−4^, at 15 K.^25^

Of particular concern in many low-temperature measurements is the dc resistance and rf loss, which can create ohmic heating and degradation of quantum information. Therefore, we characterized both types of transport properties. The measurements were conducted in both a liquid-He dewar-based system and an adiabatic demagnetization refrigerator (ADR). The samples on Si were measured with a dc 4-point probe. For the films on the 35 *μ*m thick copper traces, it was necessary to use ac transport with a lock-in amplifier due to their low resistance.

The resistivity of the sputtered-Re layers on Si was measured on ~0.6mm slices cleaved from 5mm wide strips. *T*_*c*_ taken was to be the first inflection point in the R vs. T curves. Samples were contacted by either wirebonding (25 *μ*m Al wire for low current measurements) or soldering (standard Cu wire for high current measurements). All samples could be soldered using 60:40 Sn-Pb. No problems were encountered wirebonding to the thick Cu, whereas for Pd- and Aucapped samples, Al-wirebonds did not adhere as well.

Turning to the data, for the sputter-deposited Re samples, we see a sharp transition to zero resistance in Fig. 1(a–1), with *T*_*c*_ slightly exceeding that of the highly strained Re from Ref. 10. This can be attributed to Au-Re interfacial strain that tends to expand the Re unit cell. Subsequent high temperature annealing, up to 400 °C, of this sample showed that *T*_*c*_ is stable within 0.1 K. This is in line with the high melting temperature of Re and its immiscibility with Au.^26–28^

More surprisingly, for the electroplated samples on Si, a progression of *T*_*c*_ up to approximately 6 K was obtained. First, with the Re/Au bilayer sample in Fig. 1(a–2), we measured a range of transition temperatures from *T* = 4.3 K to 4.7 K. These samples tend to tarnish over a period of a few weeks due to the exposed Re surface. Therefore, we moved to capping the samples with electroplated Au films. This resulted in more stable films and reproducible *T*_*c*_ measurements. Moreover, *T*_*c*_ in these layered samples increased to *T*_*c*_ > 5 K, with multiple steps in the transition in some samples, as shown in Fig. 1(a–3). Experiments on these films, including sputtering the top Au film off and using much thicker Au layers, tend to depress the *T*_*c*_. Subsequent samples grown as multilayers on Si, Fig. 1(a–4), demonstrated even higher enhancement of *T*_*c*_. Thinner Re films were used in the multilayers because we observed that the 75–200 nm range gave sharper transitions. Thicker films occasionally had broad transitions with multiple steps.

We then looked at the multilayered films on commercial circuit boards, Fig. 1(a–5). Here, as *T* decreases, we see the first drop in the resistance for *T* ≈ 6.1 K. However, there are slight steps in these transitions with low resistance tails. The tails go down an additional 0.1–0.5 K, depending on the current, before the transition to zero resistance.

In light of the changes of *T*_*c*_(Re) due to strain, as outlined above, the properties of the Re grown with a series of metal films, i.e., Cu, Au, and Pd, were compared. These metals all share a close-packed structure, fcc, which is similar to the hcp structure of Re. However, the near-neighbor distance in the close-packed plane for these three is 0.256, 0.275, and 0.288 nm, respectively.^29^ This spans compressive, low, and expansive strain relative to the 0.274 nm near-neighbor spacing for elemental Re.^29^ As shown in Fig. 1(b–1–3), all three types of multilayered films demonstrate enhancement of *T*_*c*_. Both Re/Au and Re/Cu have a sharp drop in resistance well above 6 K, whereas the Re/Pd samples have a slightly lower, more rounded transition. This may be due to the fact that Re, Cu, and Au are immiscible, whereas Re-Pd tends to alloy.^27^

The versatility in connecting to the samples by soldering and wirebonding was important. This allowed us to achieve good thermalization while high current densities, *J*, are applied through the soldered contacts. In the same measurement, voltage leads could be wirebonded, as shown in the inset of Fig. 2. For samples on Si substrates, we measured critical current *J*_*c*_ ≈ 2.5 to 5 × 10^8^ A/m^2^ for trilayers and multilayers, respectively. For films on Cu/PTFE, data in Fig. 2, we saw lower *J*_*c*_ ~ 10^7^ A/m^2^ due to the low-temperature tails.

While it is significant that the resistance drops to zero, it does not unequivocally demonstrate that the films are superconducting over their entire area. Magnetic measurements, which access two dimensions, are able to distinguish this behavior. For this study, the Au/(AuRe) × 10/Si multilayer film, Sample 4, was cleaved into a rectangular sample, 5.0 mm × 5.6 mm, for measurement in a magnetometer based on a superconducting quantum interference device (SQUID). In magnetic measurements of superconductors, the apparent magnetic moment is due to the field produced by shielding currents and trapped magnetic flux. The moment was measured in a perpendicular field of *μ*_0_*H* = 1 mT as a function of increasing temperature after cooling in zero field (ZFC) and after cooling in a field (FC) of *μ*_0_*H* = 1 mT, Fig. 3(a). The critical temperature, *T*_*c*_ = 5.4 K, is the end point of a broad transition from the shielding state to the normal state seen in the ZFC curve. The almost zero FC moment for *T* < *T*_*c*_ is indicative of an incomplete Meissner effect (incomplete expulsion of magnetic flux upon cooling below *T*_*c*_) due to strong flux pinning.

A hysteresis loop of magnetic moment as a function of field was measured at *T* = 1.8 K, Fig. 3(b). The shape of the loop is characteristic of type-II superconductors. The magnetic moment approaches zero at an upper critical field *μ*_0_*H*_*c*2_ ≈ 2.5 T. Symmetrical flux jumps are evident in the descending branches of the loop. A series of minor loops (not shown) with progressively higher maximum fields indicates the onset of hysteresis and therefore flux penetration and pinning, below only 0.4 mT. This is likely due to the large demagnetizing factor for the film in the perpendicular field.^30^

For most superconducting applications, notably SQUID magnetometry and quantum computing, it is also important to establish that the films maintain low loss at rf into the gigahertz regime. Therefore, resonator loss tangent tan *δ* = 1/*Q*_*T*_ vs. temperature was compared for Au/Re plated (Sample 5) vs. bare copper traces. Here, *Q*_*T*_ is the total quality factor of the resonator. The samples were placed in a magnetically shielded environment and cooled to low temperature. The rf measurements were made using a vector network analyzer. *Q*_*T*_ was determined using a Lorentzian fit^31^ to the resonance from a 1 mm wide, 18.2 mm long, grounded-coplanar-waveguide resonator on a 0.59 mm thick PTFE board. The width and gap of the resonators were 0.81 and 0.076 mm, respectively. The coupling quality factor of the resonator to the feedline *Q*_*C*_ ~ 2 × 10^4^. Because *Q*_*T*_ was significantly lower than *Q*_*C*_, the internal loss tangent tan *δ*_*i*_ = 1/*Q*_*i*_ ≈ 1/*Q*_*T*_, where *Q*_*i*_ is the internal quality factor. As shown in Fig. 4, for the bare copper board, tan *δ*_*i*_ ≈ 2 × 10^3^ for *T* < 8 K. Below about *T* = 250 mK, a slight increase appears. This increase is consistent with two-level system (TLS) loss,^32^ most likely in the PTFE.

For the Re/Au electroplated circuit board, on the other hand, a decrease in tan *δ*_*i*_ for *T* < 5 K is seen in Fig. 4. This is expected in the case of a superconducting transition in the Re-Au plating. The loss drops to that of PTFE at low temperature,^25^ and the TLS increase appears again at very low temperature. This shows that the loss is limited by the material of the circuit board and that including a layer of Re in traces will significantly improve performance at rf as well as dc.

In conclusion, this study shows that electroplated Re is superconducting with an enhanced *T*_*c*_ ≈ 6 K. This enhancement occurs when the Re is adjacent on both sides with noble metals, making it compatible with standard Cu and Au plated circuitry. It is weakly dependent on the type of metal, indicating that symmetry breaking at the interfaces likely plays a role along with possible bulk effects such as hydrogen incorporation and nano-structures.^21,24,33^ This is corroborated by ongoing studies (not shown) of time-of-flight secondary-ion mass spectroscopy, which reveals high concentrations of H in the Re, and scanning transmission electron microscopy, which shows electron transmission through the Re layers with a striated pattern perpendicular to the plane of the film. Our own density-functional-theory calculations confirm the immiscibility of Au/Re and point to the possibility of an increase in the density of states near *E*_*F*_ as the Re lattice expands, in agreement with Ref. 13. These ongoing studies may help identify the relationship between morphology and superconductivity in Re.

We had helpful discussions with Xingzhong Li, Tom Ohki, Jerry Chow, Gene Hilton, Kyle McKay, and Bahman Sarabi. We thank Paul Blanchard and Chunsheng Tian at EAG Laboratories for preparing samples and measuring TOF-SIMS. This work was supported by the Intelligence Advanced Research Projects Activity (IARPA) LogiQ Program and the NIST Quantum Based Metrology Initiative. The use of the Center for Nanoscale Materials, an Office of Science user facility, was supported by the U.S. Department of Energy, Office of Science, Basic Energy Sciences, under Contract No. DE-AC02-06CH11357. This work is a contribution of NIST, not subject to copyright.

## Figures and Tables

**FIG. 1.**
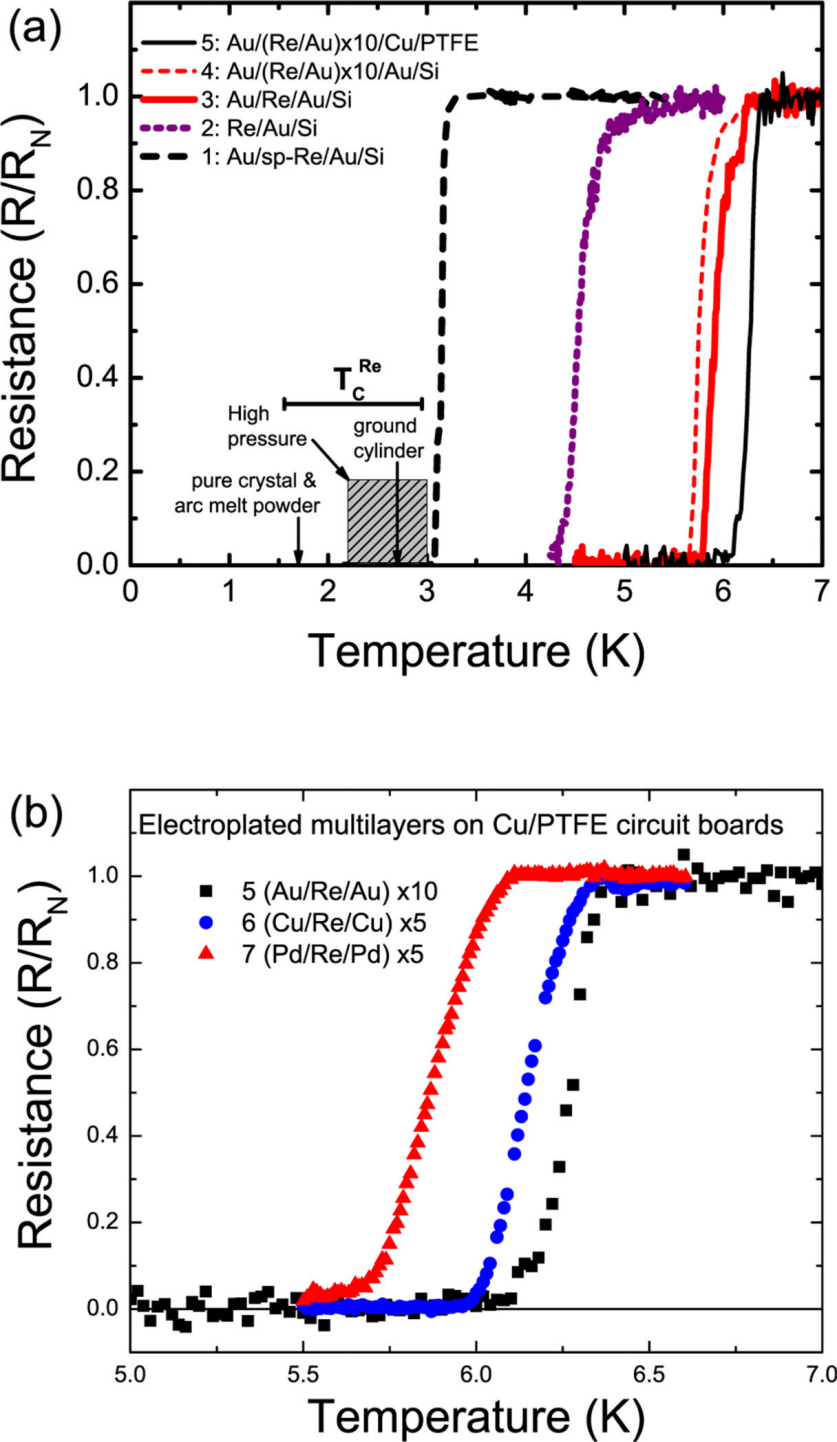
Resistance vs. temperature. (a) Data from Refs. 10 and 13 show *T*_*c*_(Re) from the literature, curves show data from Au/Re samples described in Table I; (b) comparison of critical behavior for Re in Cu, Au, and Pd multilayers, Samples 5–7.

**FIG. 2.**
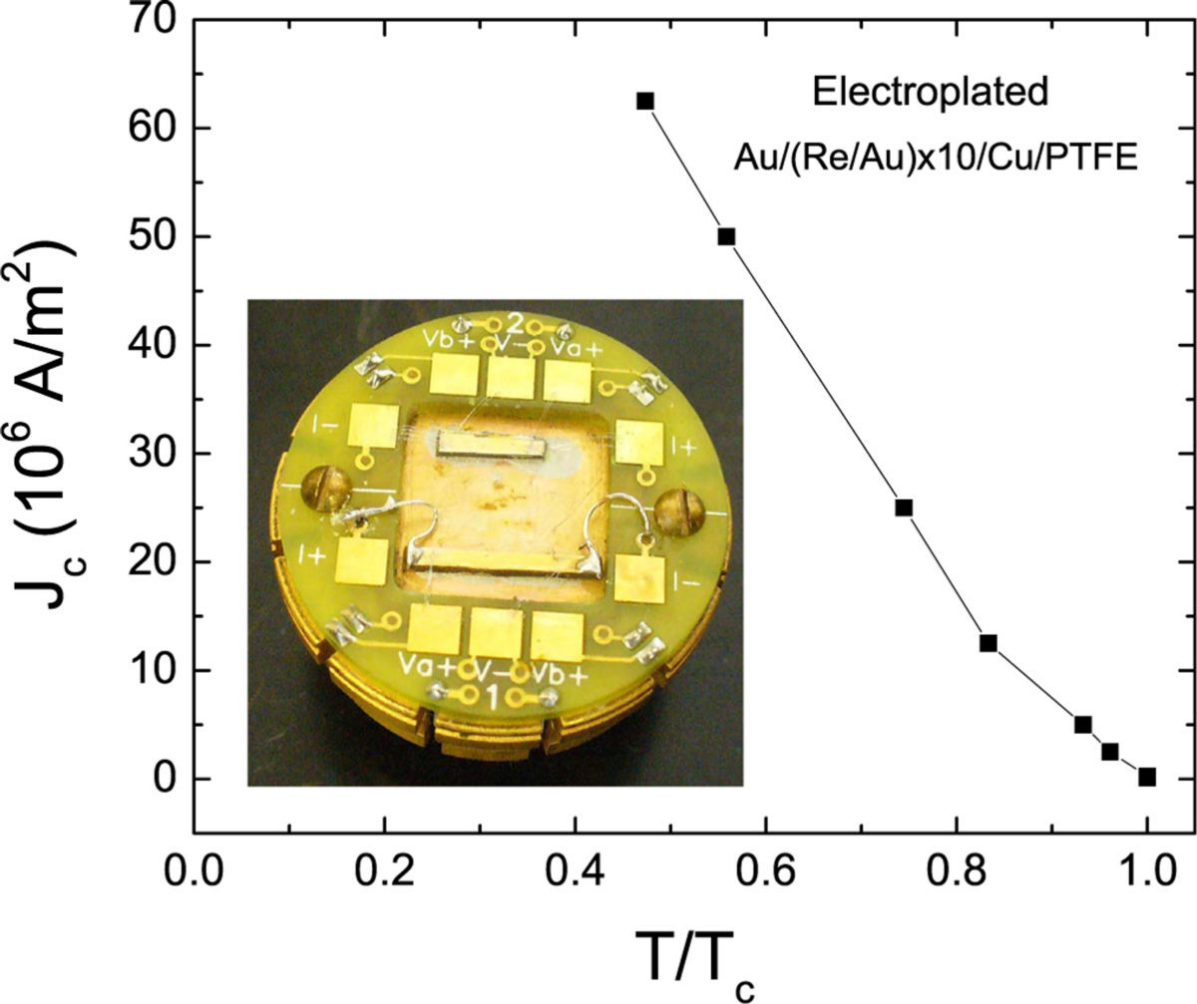
Critical current *J*_*c*_ vs. normalized temperature *t* = *T/T*_*c*_ for as-prepared electroplated multilayer, (Au/Re) × 10/Au/PTFE, Sample 5. The inset shows two samples mounted; bottom sample with current leads soldered for high currents and voltage leads wirebonded; top sample is wirebonded on both current and voltage leads.

**FIG. 3.**
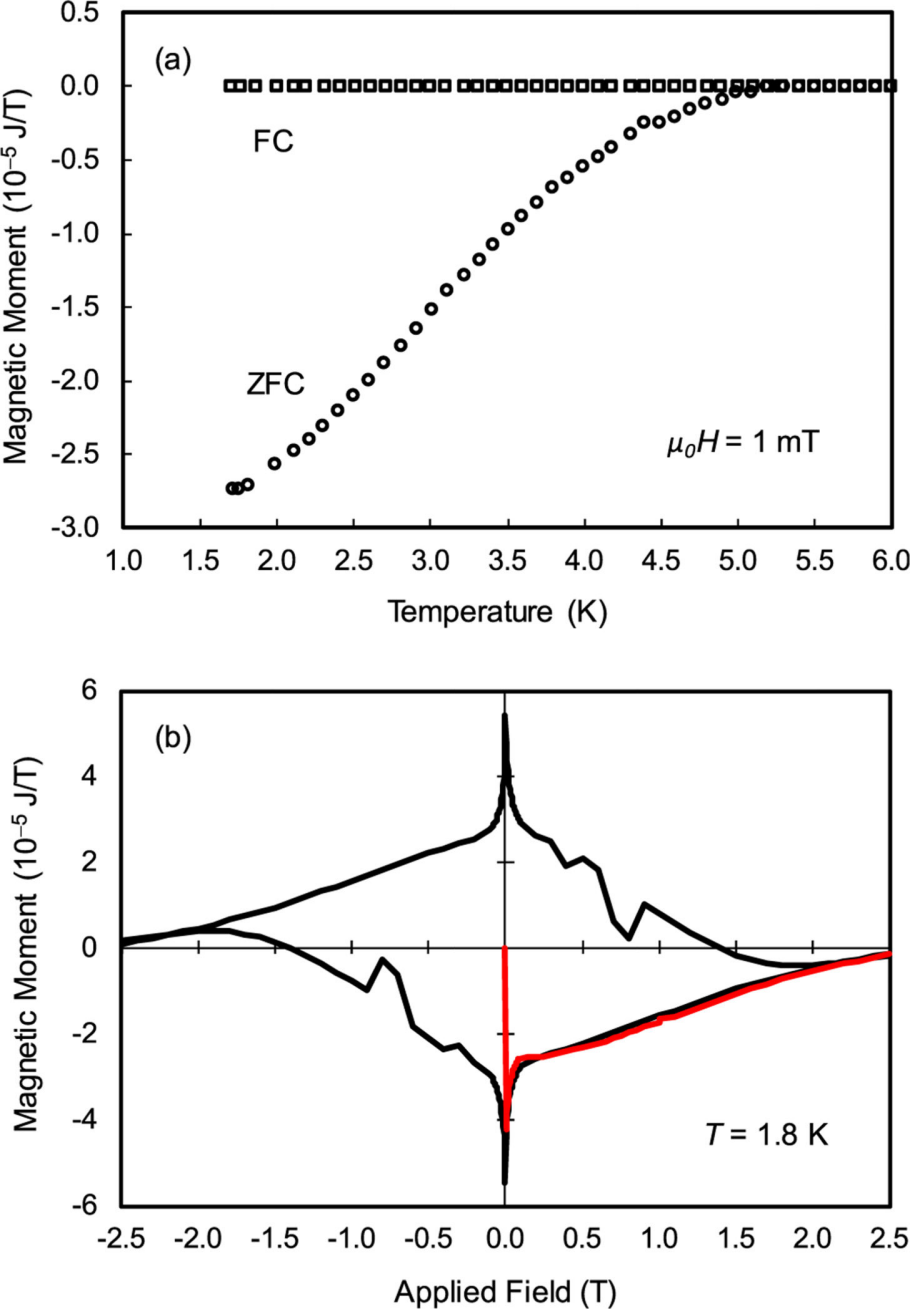
(a) Zero-field cooled (ZFC) and field-cooled (FC) magnetic moment as functions of increasing temperature measured in *μ*_0_*H* = 1.0 mT. The critical temperature is *T*_*c*_ = 5.4 K. (b) Magnetic hysteresis loop measured at *T* = 1.8 K. The initial curve with increasing field is in red. The upper critical field is *μ*_0_*H*_*c*2_ ≈ 2.5 T. Flux penetration occurs below 0.4 mT.

**FIG. 4.**
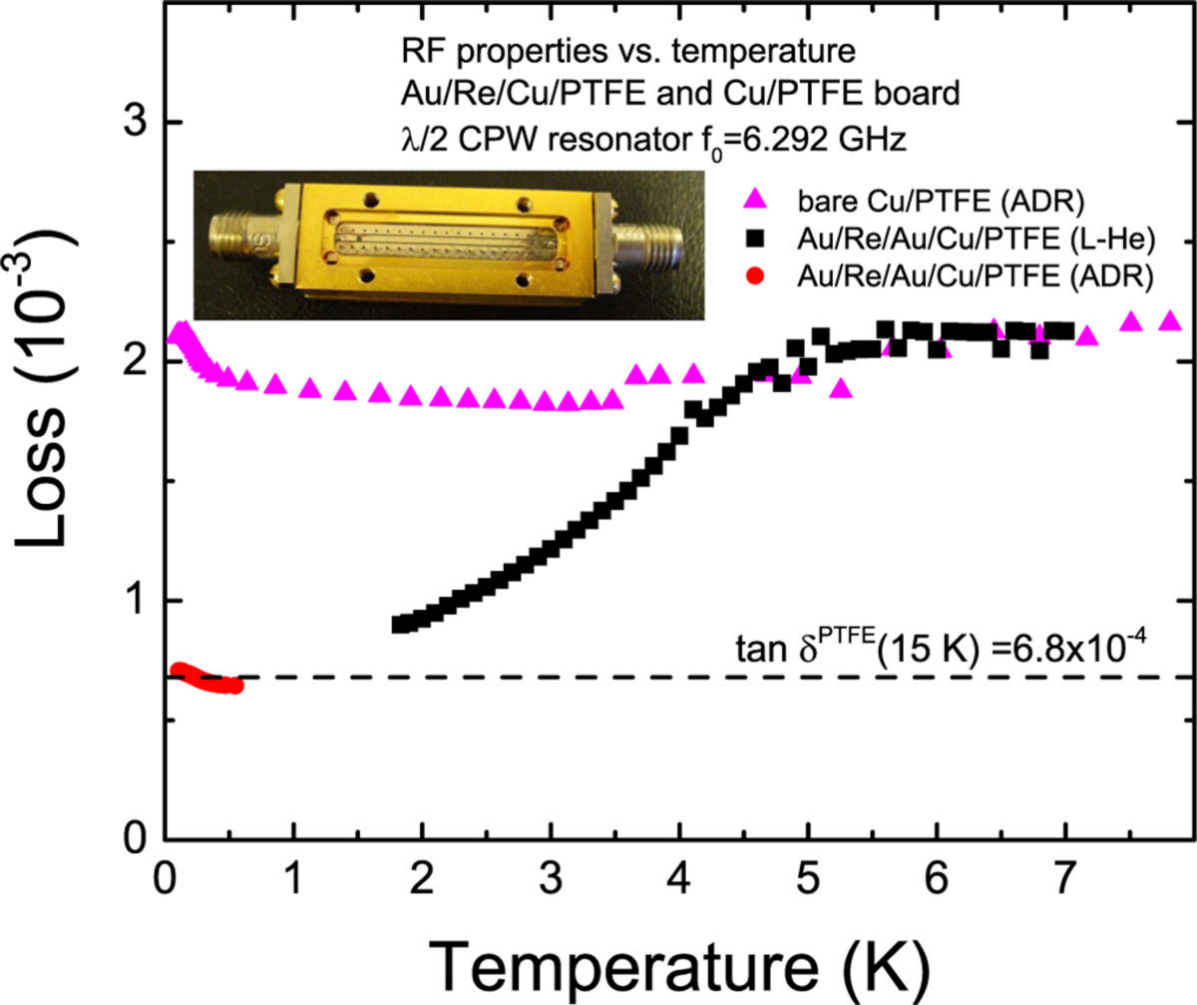
Quality factor of Au/(Re/Au) × 10/Cu/PTFE (Sample 5) compared to bare Cu/PTFE on a grounded coplanar resonator. The inset shows a Au/Re circuit board soldered into a Au-plated Cu box connectorized with SMA ports.

**TABLE I.** Sample composition and thicknesses (nm). For Sample 1, a quartz crystal thickness monitor was used. For Samples 3 and 4, thicknesses were obtained directly from STEM. Nominal thicknesses are given for Samples 2, 5, and 6 based on times derived from 3 and 4. Au, Cu, and Pd were grown using standard electroplating solutions with the exceptions being the Au metallization with a 5nm Ti adhesion layer on the Si in Samples 2–4, and the vacuum prepared trilayers for Sample 1.

_Sample_╲^Layer^	Bottom	Middle	Top

1. Vacuum deposited trilayer on Si	200 Au	300 Re	100 Au
2. Electroplated bilayer on Si	200 Au	300 Re	n/a
3. Electroplated trilayer on Si	200 Au	300 Re	75 Au
4. Electroplated multilayer on Si	200 Au	(20 Au + 75 Re) × 10	75 Au
5. Electroplated multilayer on Cu/PTFE	200 Au	(20 Au + 75 Re) × 10	75 Au
6. Electroplated multilayer on Cu/PTFE	N/A	(500 Cu + 75 Re) × 5	500 Cu
7. Electroplated multilayer on Cu/PTFE	N/A	(500 Pd + 75 Re) × 5	500 Pd

## References

[1] Mukhanov OA, IEEE Trans. Appl. Supercond 21, 760 (2011).

[2] Herr QP, Herr AY, Oberg OT, and Ioannidis AG, J. Appl. Phys 109, 103903 (2011).

[3] Manheimer MA, IEEE Trans. Appl. Supercond 25, 1301704 (2015).

[4] Holmes DS, Ripple AL, and Manheimer MA, IEEE Trans. Appl. Supercond 23, 1701610 (2013).

[5] Kotsubo V, Radebaugh R, Hendershott P, Bonczyski M, Wilson B, Nam SW, and Ullom JN, IEEE Trans. Appl. Supercond 27, 9500405 (2017).

[6] Radebaugh R, Phys J.: Condens. Matter 21, 164219 (2009). 10.1088/0953-8984/21/16/164219 21825399

[7] Devoret MH and Schoelkopf RJ, Science 339, 1169 (2013). 23471399 10.1126/science.1231930

[8] Reilly DJ, npj Quantum Inf. 1, 15011 (2015).

[9] Haynes WM, CRC Handbook of Chemistry and Physics, 92nd ed. (CRC Press, USA, 2011).

[10] Mito M, Matsui H, Tsuruta K, Yamaguchi T, Nakamura K, Deguchi H, Shirakawa N, Adachi H, Yamasaki T, Iwaoka H , Sci. Rep 6, 36337 (2016). 27811983 10.1038/srep36337 PMC5095657

[11] Alekseevski NE%Mikheeva K,MN, and Tulina NA, Sov. Phys. JETP 25, 575 (1967).

[12] Daunt JG and Smith TS, Phys. Rev 88, 309 (1952).

[13] Chu CW, McMillan WL, and Luo HL, Phys. Rev. B 3, 3757 (1971).

[14] Testardi LR, Hauser JJ, and Read MH, Solid State Commun. 9, 1829 (1971).

[15] Sundar S, Banik S, Chandra LSS, Chattopadhyay MK, Ganguli T, Lodha GS, Pandey SK, Phase DM, and Roy SB, J. Phys.: Condens. Matter 28, 315502 (2016). 27301550 10.1088/0953-8984/28/31/315502

[16] Weides MP, Kline JS, Vissers MR, Sandberg MO, Wisbey DS, Johnson BR, Ohki TA, and Pappas DP, Appl. Phys. Lett 99, 262502 (2011).

[17] Singh V, Schneider BH, Bosman SJ, Merkx EPJ, and Steele GA, Appl. Phys. Lett 105, 222601 (2014).

[18] Fink CG and Deren P, Trans. Electrochem. Soc 66, 471 (1934).

[19] Levi R, “Rhenium plating,” U.S. patent 2,616,840 (1952).

[20] Sadana YN and Wang ZZ, Surf. Coat. Technol 37, 419 (1989).

[21] Gamburg Yu. D., Drovosekov AB, and Puryaeva TP, Russ. J. Electrochem 51, 376 (2015).

[22] Zhulikov VV and Gamburg YD, Russ. J. Electrochem 52, 847 (2016).

[23] Chang S-Y, Liang L-P, Kao L-C, and Lin C-F, J. Electrochem. Soc 162, D96 (2015).

[24] Gor’kov LP, Rev. Mod. Phys 90, 011001 (2018).

[25] Mazierska J, Jacob MV, Ledenyov D, and Krupk J, in 2005 Asia-Pacific Microwave Conference Proceedings (2005), Vol. 4, p. 2370.

[26] Okamoto H and Massalski TB, Bull. Alloy Phase Diagrams 5, 383 (1984).

[27] Shunk FA, Constitution of Binary Alloys, Second Supplement (McGraw-Hill Book Company, USA, 1985).

[28] Predel B, Au-Re (Gold-Rhenium) (Springer Berlin Heidelberg, Berlin, Heidelberg, 1991), p. 1.

[29] Kittel C, Introduction to Solid State Physics, 5th ed. (John Wiley & Sons, Inc., USA, 1976).

[30] Tinkham M, Introduction to Superconductivity, 2nd ed. (Dover Publications, Mineola, New York, 1996).

[31] Probst S, Song FB, Bushev PA, Ustinov AV, and Weides M, Rev. Sci. Instrum 86, 024706 (2015). 25725869 10.1063/1.4907935

[32] Pappas DP, Vissers MR, Wisbey DS, Kline JS, and Gao J, IEEE Trans. Appl. Supercond 21, 871 (2011).

[33] Syed HM, Webb C, and Gray EM, Prog. Solid State Chem. 44, 20 (2016).

